# Effect of lidocaine and magnesium sulfate on rocuronium onset time: a randomized controlled experimental study

**DOI:** 10.1016/j.bjane.2025.844696

**Published:** 2025-10-24

**Authors:** Michele Cocenza Varrichio Crispim, Denise Aya Otsuki, Yuri Ferreira Vicentini, Danielly Roberta Penedo Rodrigues, Milena Gomes Parzianello Egúsquiza, José Otavio Costa Auler Junior

**Affiliations:** a Hospital das Clínicas da Faculdade de Medicina da Universidade de São Paulo (FMUSP), Laboratório de Investigação Médica n-8 (LIM-8), São Paulo, SP, Brazil; b Faculdade de Medicina Veterinária e Zootecnia da Universidade de São Paulo (FMVZ/USP), São Paulo, SP, Brazil

**Keywords:** Airway management, Anesthetic adjuvants, Neuromuscular blockade, Neuromuscular monitoring, Tracheal intubation

## Abstract

**Background:**

Neuromuscular blockers such as succinylcholine are widely used for airway management in critically ill patients; but their use may be contraindicated due to adverse effects. In rapid sequence intubation, the onset time of the neuromuscular blocker is critical and should be as short as possible. This study investigates whether lidocaine and magnesium sulfate could reduce the onset time of rocuronium bromide in an experimental model.

**Method:**

Eighteen animals were randomly assigned to three groups and treated with lidocaine, magnesium sulfate, or saline before receiving rocuronium bromide (3 mg.kg^-1^). After 10 minutes of neuromuscular blockade, reversal was performed with sugammadex (9 mg.kg^-1^). Onset and reversal times were measured by acceleromyography. Doses were standardized in a pilot study with four animals. Data were tested for normality using the Shapiro-Wilk and Anderson-Darling tests. Onset times are tested with a one-way ANOVA, followed by Fisher’s (LSD) post hoc test, and mean arterial pressure and heart rate with a two-way ANOVA, followed by Tukey’s post hoc test. Statistical significance was set at p ≤ 0.05.

**Results:**

The results showed that lidocaine and magnesium sulfate significantly reduced the onset time of rocuronium bromide compared to the saline solution (p < 0.05) and did not affect the onset time of reversal with sugammadex (p > 0.05). Both adjuvants caused hypotension, with a more significant effect observed with magnesium sulfate; however, blood pressure returned to baseline values.

**Conclusion:**

In conclusion, lidocaine and magnesium sulfate facilitate airway access by reducing the onset time of rocuronium bromide.

Animal Ethics Committee approved 1749/2022.

## Introduction

Endotracheal intubation for airway protection plays a crucial role in various settings, including pre-hospital care, emergency rooms, operating rooms, and intensive care units.^1^ Neuromuscular blockers are essential for tracheal intubation in situations involving the risk of gastric content aspiration or respiratory failure with severe hypoxemia.^2^ Their selection must be based on a thorough understanding of the drug's pharmacological properties, as well as the specific characteristics of each patient and clinical scenario.^3^

Although widely used, succinylcholine has several adverse effects, leading to the search for new non-depolarizing agents. Rocuronium bromide provides optimal intubation conditions within 60 seconds when administered at a dose of 1.0 mg.kg^-1^.^3^^,^^4^ It acts by competing for nicotinic cholinergic receptors at the motor endplate and can have its effect reversed by a specific agent or anticholinesterase drugs. Rocuronium is the preferred alternative when succinylcholine is contraindicated.^3^

To further shorten the onset time of rocuronium, the investigation of adjuvant drugs is warranted. Clinically established agents such as lidocaine and magnesium sulfate have been suggested to enhance neuromuscular blockade. Magnesium exerts presynaptic effects mainly through calcium interaction, inhibiting acetylcholine release, whereas lidocaine can bind to specific sites on acetylcholine receptors, leading to receptor desensitization and post-synaptic blockade. Therefore, this study aimed to evaluate the influence of lidocaine and magnesium sulfate on the onset time of rocuronium bromide, as well as its reversal with sugammadex, using Train-Of-Four (TOF) monitoring with acceleromyography in an experimental model.

## Materials and methods

The study was approved by the Animal Ethics Committee of the Faculty of Medicine of the University of São Paulo (FMUSP) under protocol number 1749/2022. It was conducted at the Medical Investigation Laboratory 08 (LIM-08) of the same institution. All procedures were performed in accordance with the Brazilian guidelines for the care and use of laboratory animals and were reported according to the ARRIVE guidelines. Animal Ethics Committee approved 1749/202.

### Animals

A total of 22 pigs, weighing between 25–30 kg, were used. They were sourced from commercial farms previously selected for their high sanitary standards. Physical and laboratory examinations were performed beforehand, and exclusion criteria included plasma hemoglobin levels below 9 mg.dL^-1^, abnormal baseline blood gas values, and clinical signs of infection. After selection, the animals underwent a 12-hour fasting period with free access to water prior to the procedure.

### Anesthetic procedure

The animals were sedated with intramuscular ketamine (5 mg.kg^-1^) combined with midazolam (0.25 mg.kg^-1^). After 15 minutes, the marginal ear vein was catheterized. An intravenous dose of 5 mg.kg^-1^ propofol was then administered for anesthesia induction. Following orotracheal intubation, the animals were placed in the supine position and mechanically ventilated (Servo-i – Maquet, Sweden) in volume-controlled mode with a tidal volume of 8 mL.kg^-1^, a respiratory rate adjusted to maintain normocapnia, an inspired oxygen fraction of 0.40, and zero Positive End-Expiratory Pressure (PEEP). The anesthetic plane was maintained with continuous infusion of propofol (200 mcg.kg^-1^.min^-1^), fentanyl (10 mcg.kg^-1^.h^-1^), and midazolam (0.5 mg.kg^-1^.h^-1^) and was assessed using the Bispectral Index (BIS). Once an adequate anesthetic plane was achieved, with BIS values between 40 and 60 ^5^, cardiovascular monitoring was initiated, including invasive blood pressure measurement and electrocardiography using a multiparameter monitor (NIHON KOHDEN – Japan). The study did not have humane endpoints, and no animals were excluded from the statistical analysis. At the end of experimental procedure, the animals were euthanized by deepening anesthesia (isoflurane 5%) and potassium chloride administration (19.1%, 1 mL.kg^-1^).

### Pilot study (dose determination)

To determine the appropriate dose of rocuronium bromide, aiming for a 90% reduction in T1 in the TOF monitoring using Acceleromyography (AMG), a pilot study was conducted with four animals. The rocuronium bromide dose required in pigs has been reported to be approximately 7-fold higher than in humans.^6^ Therefore, in the pilot study, different doses were tested to establish the onset time of rocuronium bromide in pigs. The onset times obtained were: 9’10” at 0.6 mg.kg^-1^, 2’40” at 1.2 mg.kg^-1^, 4’23” at 2.4 mg.kg^-1^ and 1’40” at 3 mg.kg^-1^.

In the same four animals, after 5 minutes of neuromuscular blockade, sugammadex was administered in varying doses (2 mg, 5 mg, 7 mg, and 9 mg) to achieve blockade reversal, targeting a T4/T1 ratio of ≥ 0.9 within 5 minutes using AMG. Only the 9 mg.kg^-1^ dose of sugammadex successfully reversed the effects of 3 mg.kg^-1^ of rocuronium bromide, maintaining a TOF ratio of 0.9 with a 5% variation in response over 2 minutes.

With respect to lidocaine and magnesium sulphate dosing, no studies employing these agents in pigs were identified in the literature. Therefore, the upper limits of the commonly reported dosage ranges were selected: 1.5–2 mg.kg^-1^ for lidocaine and 30–50 mg.kg^-1^ for magnesium sulphate.

### Neuromuscular blockade monitoring

Neuromuscular blockade monitoring was performed using the TOF module of Nihon Kohden (model AF-101P). The acceleration transducer was positioned on the facial nerve (orbicularis oculi muscle) to quantitatively assess muscle response to electrical stimulation from the transducer.

The TOF module was calibrated before each administration of rocuronium bromide, with a sensitivity range of 0–812 and an electrical current range of 0–60 mA, allowing automatic calculation of the supramaximal stimulation current. Recalibration was performed whenever the contraction height exceeded 5%. In TOF mode, three stimuli were delivered at 20 ms intervals, followed by a fourth stimulus at 0.75s.

**Figure 1:**
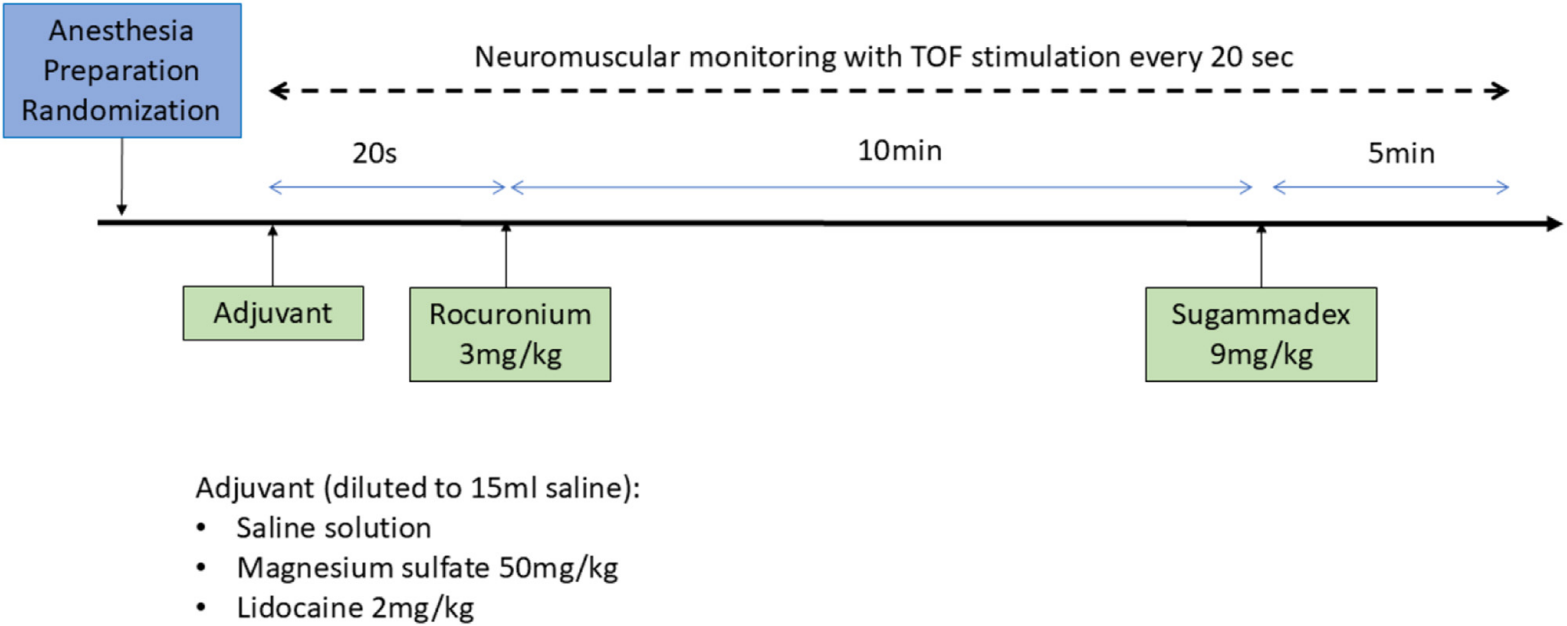
Study design.

Three baseline measurements were taken for each animal. The TOF ratio (TOFratio), TOF count (TOFcount), T1, T2, T3, and T4 values were recorded every 20 seconds for 15 minutes following calibration and baseline measurements (Fig. 1) and subsequently analyzed.

The onset time of the neuromuscular blocker was defined as the point when T1 decreased by 90% after administration, while the reversal time was recorded when T4/T1 reached 0.9 or higher after sugammadex administration. Drug onset and reversal times were precisely recorded and video-documented for further verification.

### Experimental design

This was a randomized study in which eighteen animals were allocated into three experimental groups, each consisting of six animals. Randomization was performed on the site (www.randomization.org) and the allocation list was placed in envelopes numbered 1 to 18, which were opened consecutively on the day of the experiment of each animal after preparation. Treatments were diluted to the same final volume (15 mL) by a staff member not involved in the evaluations and administered at the same speed (20 seconds) in a blinded manner.

• Saline (n = 6): Administration of 0.9% saline solution (15 mL) over 20 seconds, followed immediately by rocuronium bromide (3 mg.kg^-1^) over 5 seconds.
• Magnesium sulfate (n = 6): Administration of 10% magnesium sulfate (50 mg.kg^-1^) over 20 seconds, followed immediately by rocuronium bromide (3 mg.kg^-1^) over 5 seconds.
• Lidocaine (n = 6): Administration of 2% lidocaine (2 mg.kg^-1^) over 20 seconds, followed immediately by rocuronium bromide (3 mg.kg^-1^) over 5 seconds.

After 10 minutes of neuromuscular blockade, reversal was performed with sugammadex (9 mg.kg^-1^).

### Statistical analysis

This was an exploratory study, and no a priori sample size calculation was performed. A post hoc sample size calculation was performed based on the rocuronium onset time results obtained from groups of 6 animals each. The estimated required sample size ranged from 10 to 14 animals per group, assuming an alpha level of 0.05 and a statistical power of 0.80. With the current sample size of 6 animals per group, the calculated power was 0.41 for the comparison between saline and lidocaine and 0.55 for the comparison between saline and magnesium. We decided to keep the sample size at 6 per group, considering ethical concerns regarding animal use and the associated costs.

Normality was assessed using the Shapiro-Wilk and Anderson-Darling tests. Onset times of the neuromuscular blocker and the reversal agent were analyzed with a one-way ANOVA, followed by Fisher’s Least Significant Difference (LSD) post hoc test. Mean arterial pressure and heart rate were analyzed with a two-way ANOVA, followed by Tukey’s post hoc test. Statistical significance was set at p ≤ 0.05

## Results

The onset time for rocuronium bromide and sugammadex are presented in Table 1. Both adjuvants, lidocaine and magnesium sulfate, significantly reduced the mean onset time compared with saline [F(2,15) = 4.385, p = 0.0317]. However, no difference was observed in the reversal with sugammadex.

**Table 1:** Onset time of rocuronium (3 mg.kg^-1^) and the antagonist sugammadex (9 mg.kg^-1^), in seconds, with and without adjuvants, lidocaine (2 mg.kg^-1^), and magnesium sulfate (50 mg.kg^-1^), in an experimental model.

	Group (n = 6/group)	Onset time (sec)		ANOVA p
		Mean (SD)	95% CI	
**Rocuronium**	Lidocaine	81 (19)^a^	[60.8; 100.9]	p = 0.0317
	Magnesium	69 (19)^b^	[49.1; 88.7]	
	Saline	143 (77)	[63.2; 223.4]	
**Sugammadex**	Lidocaine	206 (35)	[170; 243]	p = 0.5976
	Magnesium	160 (87)	[69; 251]	
	Saline	207 (127)	[74; 341]	

SD, Standard Deviation; One-way ANOVA and Fisher LSD post hoc test.

a Difference between Lidocaine and Saline (p = 0.037).

b Difference between Magnesium and Saline (p = 0.016).

The Mean Arterial Pressure (MAP) and Heart Rate (HR) values are summarized in Table 2, Table 3. Differences in MAP were observed across time points in the groups that received magnesium sulfate and lidocaine. In both adjuvant groups, MAP was lower than in the saline group after rocuronium administration. No difference was observed between groups in HR.

**Table 2:** Mean arterial pressure and heart rate with the use of rocuronium (3 mg.kg^-1^) and the antagonist sugammadex (9 mg.kg^-1^), with and without adjuvants, lidocaine (2 mg.kg^-1^), and magnesium sulfate (50 mg.kg^-1^), in an experimental model.

	Group (n = 6/group)	Baseline		After adjuvant		After rocuronium		Muscular blockade		After reversion	
		Mean (SD)	95% CI	Mean (SD)	95% CI	Mean (SD)	95% CI	Mean (SD)	95% CI	Mean (SD)	95% CI
**HR**	Lidocaine	92 (20)	71; 114	98 (22)	75; 122	101 (21)	78/123	105 (21)	83; 127	103 (19)	83; 123
	Magnesium	95 (12)	82; 107	93 (16)	76; 110	93 (17)	75; 111	102 (17)	85; 119	96 (16)	79; 114
	Saline	88 (14)	73; 102	87 (16)	70; 104	96 (18)	77; 115	101 (25)	75; 127	92 (30)	71; 114
**MAP**	Lidocaine	71 (11)	59; 83	69 (11)	57; 80	63 (12)^c^	50; 76	72 (22)	49; 95	74 (11)	62; 86
	Magnesium	73 (7)	65; 80	65 (7)^c^	58; 72	58 (7)^a^^,^^c^	51; 66	68 (7)	60; 75	72 (7)^b^	65; 80
	Saline	79 (7)	70; 91	81 (10)	71; 91	86 (12)	74; 99	82 (14)	67; 96	84 (14)	70; 99

MAP, Mean Arterial Pressure; HR, Heart Rate; SD, Standard Deviation; Two-way ANOVA with Tukey post hoc test for difference between groups and time points.

a p < 0.05 vs. Basal;

b p < 0.05 vs. After rocuronium;

c p < 0.05 vs. Saline group.

**Table 3:** Two-way ANOVA summary.

	SS	DF	MS	F (DFn, DFd)	p-value
**HR**					
Interaction	365.5	8	45.69	F (8. 60) = 0.3752	p = 0.9297
Time	1442	4	360.6	F (1.826. 27.39) = 2.961	p = 0.0727
Grupo	760.8	2	380.4	F (2. 15) = 0.3038	p = 0.7425
Subject	18786	15	1252	F (15. 60) = 10.29	p < 0.0001
Residual	7305	60	121.8		
**MAP**					
Interaction	895.7	8	112.0	F (8. 60) = 2.561	p = 0.0179
Time	608.4	4	152.1	F (2.071. 31.06) = 3.479	p = 0.0419
Grupo	4058	2	2029	F (2. 15) = 4.204	p = 0.0355
Subject	7239	15	482.6	F (15. 60) = 11.04	p < 0.0001
Residual	2623	60	43.71		

MAP, Mean Arterial Pressure; HR, Heart Rate; SS, Sum of Squares; DF, Degrees of Freedom; MS, Mean Square; F, F-statistic.

## Discussion

A significant reduction in the onset time of rocuronium was observed with lidocaine and magnesium sulfate compared to saline. Regarding the reversal of the blockade, adjuvants did not significantly interfere with reversal after sugammadex administration. This approach may be valuable in situations that require rapid sequence intubation, offering an alternative to succinylcholine in scenarios where this agent is contraindicated.

The need to shorten the onset time of non-depolarizing neuromuscular blockers is crucial in urgent intubation scenarios.^7^ Translational studies are particularly valuable in this context, as they allow the evaluation of pharmacological strategies for emergency situations without exposing critically ill patients to additional risks. Although experimental doses require adjustment for clinical practice and metabolic differences exist between pigs and humans, animal models remain an essential first step for validating new treatments and interventions.

The TOF monitoring was employed to assess both onset and reversal of the blockade, in accordance with the recommendations of the American Society of Anesthesiologists (ASA), which emphasize the importance of adequate monitoring, particularly during reversal, to avoid residual blockage and mitigate individual variability.^8^

Rocuronium provides excellent conditions for intubation in an estimated time of 90 seconds, considered satisfactory for this purpose.^9^ When administered after saline solution, a more prolonged blockade onset was observed compared to magnesium and lidocaine, with a duration ranging between 80 and 240 seconds.

The main results of this study indicate that both lidocaine and magnesium sulfate were effective in reducing the onset time of the non-depolarizing neuromuscular blockade caused by rocuronium bromide. However, some limitations must be acknowledged. The small sample size may increase the risk of type II error and bias, while the homogeneity of the experimental animals could compromise the generalizability of our findings. Additionally, adverse systemic effects, such as electrolyte alterations related to magnesium, were not assessed.

In the magnesium group, the maximum blockade time was 90 seconds, which is close to the minimum time in the lidocaine group, at 80 seconds. One limitation of the study was the absence of a group combining both adjuvants; however, such a combination is not commonly used in practice.

Magnesium sulfate demonstrated a significant reduction in the onset time of rocuronium bromide, and this efficacy is attributed to possible pre- and post-synaptic mechanisms of action. By competing with calcium, magnesium reduces acetylcholine release at the neuromuscular junction, thereby facilitating the effect of neuromuscular blockers.^10^ This interaction may explain the reduced onset time observed in our study.

For lidocaine, the reduction in onset time of rocuronium bromide may be attributed to a likely post-synaptic mechanism, through binding to acetylcholine receptors, leading to receptor desensitization and transient channel blockade. Lidocaine may also impair both pre- and post-junctional nerve conduction, further enhancing neuromuscular blockade.^11^

Among the various neuromuscular monitoring modalities available in acceleromyography, TOF is highlighted for its reliability and was selected in this study, consistent with the most recent ASA guidelines.^11^

Regarding reversal with sugammadex, lidocaine and magnesium sulfate did not produce clinically relevant interference with reversal of muscle function. In a previous study,^12^ involving 125 adult patients with ASA I or II physical status, they concluded that the combination of lidocaine (1.5 mg.kg^-1^) and rocuronium at low doses (0.6 mg.kg^-1^) was equivalent to succinylcholine. The doses used in our experimental model were higher than those commonly applied in clinical practice, which limits direct extrapolation to humans. In addition, we observed a wide variability in reversal times, which may be more related to the experimental porcine model than to a true pharmacological effect. This variability, together with the limited statistical power of our study, may also have hindered the detection of potential secondary effects.

Previous studies^13^ also demonstrated that magnesium pre-treatment enhances the neuromuscular blockade effect of rocuronium, reducing its onset time without clinically significant prolongation of blockade duration, in agreement with our findings.

Regarding the reversal of neuromuscular blockade, the magnesium-treated group showed a prolonged reversal time, reaching 320 seconds. This difference may be attributed to several causes, as discussed previously.^14^ The presence of magnesium appears to enhance the effects resulting from partial occupation of post-junctional nicotinic receptors by free rocuronium molecules, leading to a longer reversal time in this specific group.

Finally, a significant reduction in blood pressure was observed in groups treated with lidocaine and magnesium, compared to saline. Lidocaine may reduce vascular resistance, while magnesium sulfate decreases intracellular calcium by acting as a calcium channel blocker, promoting vasodilation.^15^ Both mechanisms resulted in a more pronounced reduction in blood pressure in the magnesium group compared to lidocaine. Despite this, blood pressure was restored without intervention, suggesting that the hypotensive effect may have limited clinical relevance. Nonetheless, further studies are warranted to better define these hemodynamic effects and their implications.

## Conclusion

Lidocaine and magnesium sulfate were effective in reducing the onset time of the non-depolarizing blocker rocuronium bromide. This research demonstrates that both lidocaine and magnesium sulfate did not interfere with the reversal of neuromuscular blockade and reduced its onset time, presenting themselves as good alternatives for rapid access to the airways.

## Conflicts of interest

The authors declare no conflicts of interest.

## Data Availability

The datasets generated and/or analyzed during the current study are available from the corresponding author upon reasonable request.
